# Evaluating the knowledge, attitudes, and practices of healthcare professionals towards the baby-friendly hospital initiative: a cross-sectional study in Jordanian maternity hospital

**DOI:** 10.3389/fgwh.2026.1728029

**Published:** 2026-03-25

**Authors:** Lana Alnimer, Hiba Ali Almallah, Razan Mahmoud Omoush, Hebah Ali, Adam Tawfiq Amawi, Haitham Jahrami, Suhaila Halasa, Hadeel Ali Ghazzawi

**Affiliations:** 1 Department of Nutrition and Food Technology, School of Agriculture, The University of Jordan, Amman, Jordan; 2 Department of Movement Sciences and Sports Training, School of Sport Sciences, The University of Jordan, Amman, Jordan; 3 Ministry of Health, Manama, Bahrain; 4 Department of Psychiatry, College of Medicine and Medical Sciences, Arabian Gulf University, Manama, Bahrain; 5 Department of Maternal and Child Health Nursing, School of Nursing, The University of Jordan, Amman, Jordan

**Keywords:** accreditation, baby-friendly hospital initiative, healthcare professionals, KAP, maternal baby health

## Abstract

**Background:**

Knowledge, attitudes, and practices of healthcare professionals regarding the Baby-Friendly Hospital Initiative (BFHI) practices are one of the most promising means of increasing the prevalence of initiation, duration and exclusive breastfeeding. Hence, we aimed to assess healthcare professionals’ knowledge, attitude, and practices toward Baby-Friendly Hospital Initiatives practices within accredited and non-accredited Baby- Friendly Hospitals.

**Methods:**

A descriptive, cross-sectional study was performed in 7 Jordanian hospitals (3 accredited BFHI and 4 are non-accredited BFHI) consisted of 280 healthcare professionals who were working in maternity wards and providing healthcare for mothers and their newborns. The data was collected through a four-part self-reported questionnaire that consisted of: sixteen questions on socio-demographic data, twelve questions related to knowledge, eighteen questions about healthcare professionals’ attitude and thirteen questions related to the practices toward BFHI.

**Results:**

Most of the study population (accredited and non-accredited BFHI) had a good level of knowledge (96.1% and 87% respectively) and more than half had an adequate attitudes level (65% and 61% respectively). There was a statistically significant moderate positive relationship between knowledge and attitudes levels (*r* = 0.593, *p*-value <0.001. There was a significant correlation between knowledge (*P*-value = 0.01) and attitude (*P*-value = 0.007) levels and BFHI training. Furthermore, practice levels were significantly associated with gender, marital status, having children, breastfeeding/infant feeding policy available for mothers and hospital staff, and BFHI training (*p*-value <0.05).

**Conclusions:**

Healthcare professionals (HCPs) in accredited BFHI have better knowledge, attitudes, and practices than HCPs in non-accredited BFHI. Training of all HCPs in BFHI skills is necessary to implement the ten steps policy for the purpose of improving and promoting mother's and infant's health.

## 1 Introduction

The Baby-Friendly Health Initiative (BFHI) was introduced in 1991 through a joint effort by the World Health Organization (WHO) and UNICE (1). The initiative provides a framework for breastfeeding policies aimed at supporting mothers and infants in achieving successful breastfeeding, thereby enhancing their overall health outcomes (1). Central to this initiative is the requirement that healthcare professionals (HCPs) receive proper education and training on the “Ten Steps to Successful Breastfeeding,” which serve as the guiding principles of the BFHI (2). These steps are recognized as beneficial for all mothers and infants, as they foster parent–infant bonding, strengthen parental responsiveness, empower families, and promote informed decision-making (3).

Moreover, to ensure consistency of care, the BFHI emphasizes that HCPs should be knowledgeable about and adhere to these steps, as well as to the associated guidelines, in order to avoid providing conflicting advice (4). HCPs are defined as licensed professionals with specialized education who are qualified to deliver healthcare services to individuals, families, and communities (5). Within this framework, the BFHI equips HCPs and health facilities with the tools needed to provide mothers with accurate information, confidence, and practical skills to initiate and sustain breastfeeding (2). Furthermore, HCPs have a strong influence over parents and how they can guide them to make the right decisions regarding their infants (6). Although counseling by HCPs is known to improve breastfeeding initiation and duration, evidence suggests that many professionals are not adequately prepared for this responsibility. Globally, there is growing concern about the need to strengthen education on BFHI practices within health sciences curricula. In Jordan, however, there is a particular gap in research on HCPs' knowledge of BFHI principles, highlighting the urgency of investigating this issue (2, 7–11).

The World Health Organization (WHO) and UNICEF recommend initiating breastfeeding within the first hour after birth and continuing exclusive breastfeeding for four to six months, during which infants should receive only breast milk, without additional liquids or solids, including water (12). Substantial evidence confirms the health benefits of breastfeeding for both mothers and infants (13, 14).

To our knowledge, this is the first study in Jordan to evaluate healthcare professionals' (HCPs) knowledge, attitudes, and practices regarding the Baby-Friendly Health Initiative (BFHI). Assessing and improving HCPs' competence in this area is essential, as their guidance directly influences breastfeeding initiation, exclusivity, and duration issue (2, 7, 8).

Therefore, this study aims to: (1) assess HCPs' knowledge, attitudes, and practices toward BFHI principles in accredited and non-accredited baby-friendly hospitals, and (2) compare these factors across the two settings.

## 2 Methods

### 2.1 Study design

This study used a descriptive, cross-sectional design using a convenience sample of healthcare professionals (HCPs). The design was chosen to provide a snapshot of HCPs' knowledge, attitudes, and practices regarding the Baby-Friendly Health Initiative (BFHI). Participants were recruited from primary care maternity and obstetric units in seven hospitals distributed across the Hashemite Kingdom of Jordan. These hospitals were categorized into two groups: accredited and non-accredited Baby-Friendly Hospitals (BFHs).

### 2.2 Study settings

Data were collected from the primary care and obstetric units of seven hospitals across Jordan, divided into accredited and non-accredited BFHs. The accredited group included Al-Bashir Hospital, Princess Haya Military Hospital, and Al-Karak Hospital, all accredited by the Health Care Accreditation Council (HCAC). The non-accredited group consisted of Prince Rashid Ben Al-Hasan Military Hospital, Al-Salt General Educational Hospital, Al-Nadeem Hospital, and Jameel Al-Totangi Hospital. These hospitals were selected to provide a mix of accredited and non-accredited facilities, ensuring diversity in setting and enabling comparisons between the two groups.

### 2.3 Sample size and sampling method

The study targeted healthcare professionals (HCPs), including pediatricians, gynecologists, obstetricians, nurses, midwives, and nutritionists working in maternity and obstetric units of the selected hospitals. Data were collected between June 2022 and October 2022.Inclusion criteria were HCPs directly involved in maternal and infant care in maternity wards. Exclusion criteria included HCPs not engaged in maternity or obstetric care (e.g., surgeons, cardiologists, endocrinologists, otolaryngologists) and hospitals still under evaluation for accreditation by the Health Care Accreditation Council (HCAC). The sample size was determined by the availability of medical staff in each hospital. A total of 280 HCPs participated, distributed as follows: 20 from Al-Karak Governmental Hospital, 45 from Al-Bashir Hospital, 112 from Princess Haya Military Hospital, 20 from Prince Rashid Ben Al-Hasan Military Hospital, 24 from Al-Nadeem Hospital, 20 from Jameel Al-Totangi Hospital, and 36 from Al-Salt General Educational Hospital.

### 2.4 Data collection and instruments

Participants completed the questionnaire either in person using paper forms or electronically via Google Forms, accessible through a link or a QR code. The questionnaire was adapted from Daniels (2011), with validity and reliability previously established. Minor modifications were made to ensure suitability for the Jordanian healthcare context and infrastructure (15).

The final tool was a self-administered questionnaire composed of four sections: (1) sixteen items on socio-demographic characteristics; (2) twelve items assessing knowledge of Baby-Friendly Health Initiative (BFHI) principles; (3) eighteen items evaluating healthcare professionals' attitudes; and (4) thirteen items measuring practices

#### 2.4.1 Personal and socio-demographic characteristics

This section of the questionnaire collected socio-demographic information, including age, gender, marital status, number of children, occupation, workplace, and years of experience. In addition, six multiple-choice questions assessed participants' background knowledge and exposure to BFHI principles, such as awareness of hospital accreditation status, familiarity with baby-friendly hospital practices, access to breastfeeding-related publications, and prior training.

#### 2.4.2 Healthcare professionals' knowledge and attitude

Knowledge was assessed using twelve items adapted from validated instruments (16). Each item was rated on a five-point Likert scale ranging from *strongly agree* (5) to *strongly disagree* (1). Attitudes toward BFHI were measured with eighteen items, also scored on the same five-point Likert scale (*strongly agree* to *strongly disagree*)

#### 2.4.3 Healthcare professionals' practice

Practices were evaluated using thirteen items. Most were structured as yes/no questions, while selected items were presented on a five-point Likert scale to capture agreement levels. Examples of negatively worded items included: “*Marketing and distribution of free formula samples should be allowed in hospitals.*” Scores ranged from 1to 5 (Strongly agree—Strongly disagree). Positively worded items included: “*The Baby-Friendly Hospital Initiative is a global effort to implement practices that protect, promote, and support breastfeeding*” with scores ranged from 5 to 1 (Strongly agree—Strongly disagree). Negatively phrased items were reverse scored to ensure consistency in analysis.

### 2.5 Statistical analysis

Statistical analysis was carried out using the Statistical Package for Social Sciences (SPSS, version 26) under the supervision of a biostatistician. Descriptive statistics were obtained for healthcare professionals' baseline characteristics. Frequencies and percentages were calculated for the categorical data, pearson correlation coefficient (*r*) and a chi-square test (Fisher exact test) was conducted to compare the proportions of the categorical variables for HCPs' knowledge, practices, and attitude among BFHI. The level of significance was set at *p*-value ˂0.05 to test the hypothesis of no association.

## 3 Results

### 3.1 Characteristics of participants and hospitals

This study included 280 participants (63 males and 217 females) over 20 years of age. Table 1 shows the socio-demographic characteristic of the participants. About half of the participants were 31–40 years old (49.6%). Approximately two third of the participants 79.6% (*n* = 223) were married with children 77.9% (*n* = 218). Half of the participants were nurses (50.3%), and the majority of the participants had more than 5 years of experience.

**Table 1:** Characteristics of participants (*n* = 280).

Participants characteristics		
Variable		*n* (%)
Gender	Male	63 (22.5)
	Female	217 (77.5)
Age (Year)	20–30	86 (30.7)
	31–40	139 (9.6)
	>40	55 (19.7)
Marital Status	Single	57 (20.4)
	Married	223 (79.6)
Having Children	Yes	218 (77.9)
	No	62 (22.1)
Profession	Physician	49 (16.8)
	Nurse	141 (50.3)
	Nutritionists	36 (12.9)
	Midwife	56 (20)
Years of Experience	˂5	48 (17.1)
	5–10	94 (33.6)
	10–15	71 (25.4)
	˃15	67 (23.9)

BFHI, baby friendly hospital initiatives; n, number of participants; %, percentage of participants out of 100%.

### 3.2 Knowledge, attitude, and practice levels of participants

**Figure 1:**
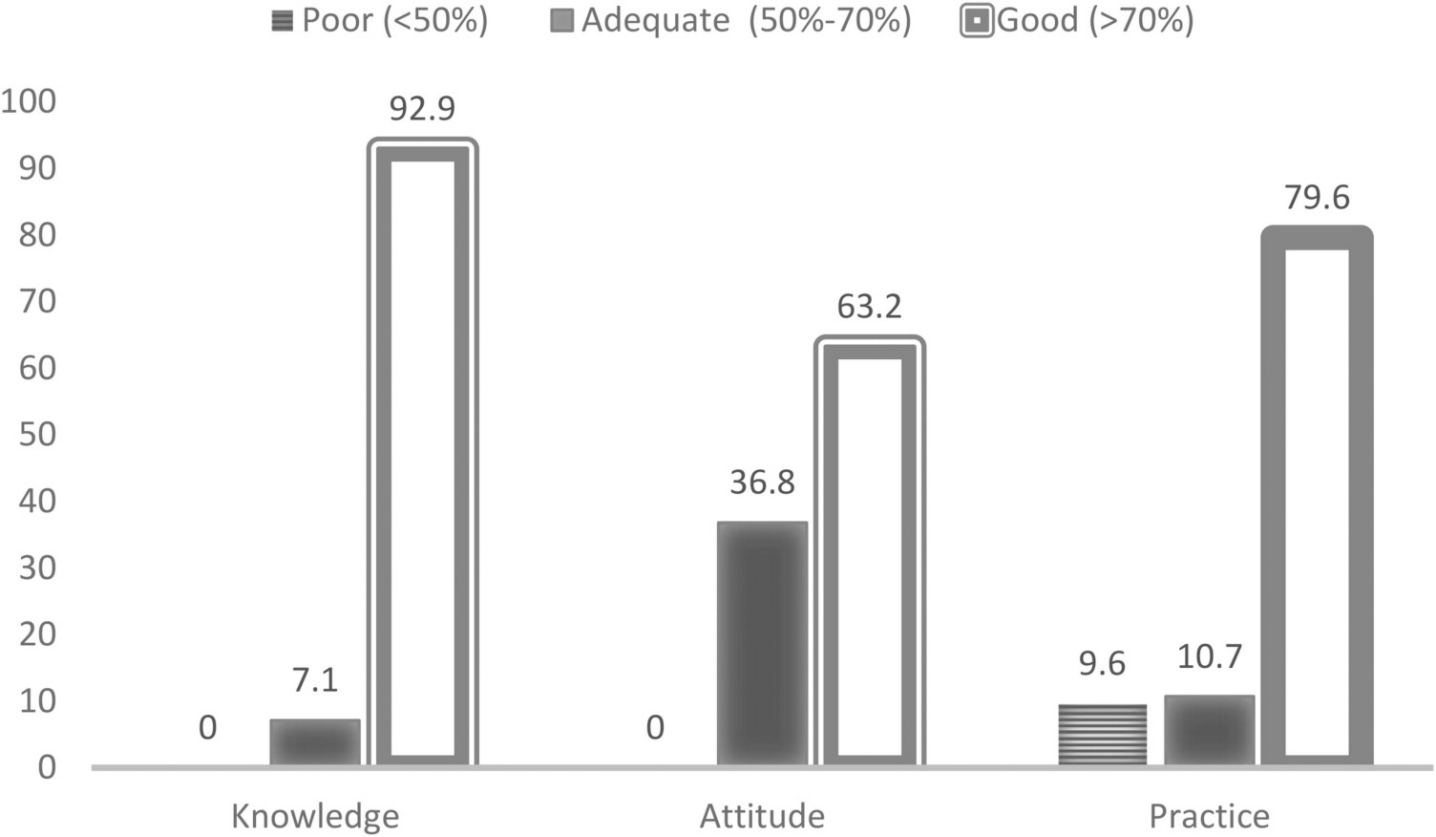
Distribution of participants among different KAP levels toward BFHI.

Figure 1 shows the knowledge, attitude, and practice of participants (*n* = 280) toward BFHI. Most of the respondents (92.9%) had good knowledge (scores more than 70%), giving the correct answer about BFHI with a mean score of 87.1% (SD = 10.8).

More than half of the participants had a good attitude (scores more than 70%) toward BFHI 63.2%, and 36.4% had an adequate level (scores of 50%–70%) of attitude and no participants had a poor level (scores lower than 50%) of attitude where they provided the correct answer about BFHI with a mean score of 74% (SD = 8.4).

Furthermore, about two third of the participants (79.6%) had good practice toward BFHI, while only 10.7% and 9.6% had adequate and poor practice toward BFHI respectively, in which they provided the correct answer about BFHI with a mean score of 84.6% (SD = 23.7).

### 3.3 Knowledge, attitudes, and practice toward BFHI regarding healthcare professionals' categories

As shown in Table 2 there were no significant differences in the level of knowledge, attitudes, and practice of HCPs toward BFHI (*p*-value ˃0.05).

**Table 2:** Distribution of participants among different KAP categories according to type of hospitals and HCPs.

KAP	Type of hospitals			HCPs				
	BFH *n* (%) *n* (180)	Non-BFH *n* (%) *n* (100)	*p*-value	Physician *n* (%) *n* (47)	Nurses *n* (%) *n* (141)	Nutritionist *n* (%) *n* (36)	Midwife *n* (%)n (56)	*p*-value
Knowledge			0.005*					0.128
Poor (<50%)	0	0		0	0			
Adequate (50%–70%)	7 (3.9)	13 (13.0)		1 (2.1)	15 (10.6)	1 (2.8)	3 (5.4)	
Good (>70%)	173 (96.1)	87 (87.0)		46 (97.9)	126 (89.4)	35 (97.2)	53 (94.6)	
Attitude			0.567					0.337
Poor (<50%)	0	0		0	0			
Adequate (50%–70%)	46 (35.6)	39 (39.0)		17 (36.2)	58 (41.1)	11 (30.6)	16 (28.6)	
Good (>70%)	116 (64.4)	61 (61.0)		30 (63.8)	83 (58.9)	25 (69.4)	40 (71.4)	
Practice			0.413					0.453
Poor (<50%)	18 (10.0)	9 (9.0)		5 (10.6)	13 (9.2)	5 (13.9)	4 (7.1)	
Adequate (50%–70%)	16 (8.9)	14 (14.0)		7 (14.9)	14 (9.9)	6 (16.7)	3 (5.4)	
Good (>70%)	146 (81.1)	77 (77.0)		35 (74.5)	114(80.9)	25(69.4)	49(87.5)	

* *p*-value <0.05, n, number of participants; %, percentage of participants out of 100.

In terms of knowledge, most of the physicians, nurses, nutritionists, and midwives had a good level of knowledge about BFHI (97.9%, 89.4%, 97.2%, and 94.6% respectively), while none of the healthcare workers had poor knowledge, regardless of their professions. Whereas, in terms of attitudes, about 63.8% of physicians, 58.9% of nurses, 69.4% of nutritionists, and 71.4% of midwives had a good level of attitude about BFHI, while none of the healthcare workers had a poor attitude, regardless of their professions.

It was found that two third of physicians (*n* = 35) 74.5% and nutritionists (*n* = 25) 69.4% had a good level of practice and most nurses and midwives had a good level of practice (80.9, 87.5, respectively). Overall, a small percentage of physicians (10.6%), nurses (9.2%), nutritionists (13.9%), and midwives (7.1%) had a poor level of practice toward BFH.

### 3.4 Correlation between demographic variables and knowledge, attitude, and practice levels

Table 3 represent the association between variables and levels of KAP survey for all participants regardless of the type of hospital, and the results showed that there was no significant association between knowledge, attitude levels and demographic variables except BFHI training. The relationship between knowledge, attitude levels and BFHI training was significant. Participants who attended BFHI training were more likely to have good knowledge and attitude levels. Practice levels were significantly associated with most demographic variables including gender, marital status, having children, breastfeeding/infant feeding policy available for mothers and staff in hospitals, and BFHI training. Other variables including age, profession and years of experience were not significantly associated with the practice levels of participants.

**Table 3:** Distribution of participants Among different KAP categories according to their demographic characteristics.

Variables	Knowledge Level *n* (%)			*P*-value
	Adequate (50%–70) 20 (7.1)		Good (>70%) 260 (92.9)	
Training regarding BFHI				
Yes	1 (5.0)		84 (32.3)	0.010*
No	19 (95.0)		176 (67.7)	
Variables	Attitude Level *n* (%)			*P*-value
	Adequate (50%–70%) 102 (36.8)		Good (>70%) 178 (63.2)	
Training regarding BFHI				
Yes	21 (20.6)		64 (36.0)	0.007*
No	81 (79.4)		114 (64.0)	
Variables	Practice levels *n* (%)			
	Poor (<50%) 27 (9.6)	Adequate (50%–70%) 30 (10.7)	Good (>70%) 223 (79.6)	
Sex				
Male	7 (25.9)	12 (40.0)	44 (19.7)	0.040*
Female	20 (74.1)	18 (60.0)	179 (80.3)	
Marital status				
Single	6 (22.2)	11 (36.7)	40 (17.9)	0.05*
Married	21 (77.8)	19 (63.3)	183 (82.1)	
Having Children				
Yes	18 (66.7)	19 (63.3)	181 (81.2)	0.029*
No	9 (33.3)	11 (36.7)	42 (18.8)	
Breastfeeding/infant feeding policy available for all staff in hospital				
Yes	14 (51.9)	16 (53.3)	178 (79.8)	0.000*
No	13 (48.1)	14 (46.7)	45 (20.2)	
Breastfeeding/infant feeding policy available for mothers in hospital				
Yes	16 (59.3)	17 (56.7)	176 (78.9)	0.005*
No	11 (40.7)	13 (43.3)	47 (21.1)	
Training regarding BFHI				
Yes	2 (7.4)	3 (10.0)	80 (35.9)	0.000*
No	25 (92.6)	27 (90.0)	143 (64.1)	

*p-value <0.05. for supplementary clarify detailed material are in appendix number, n: number of participants, %: percentage of participants out of 100%.

### 3.5 Correlation between knowledge, attitudes, and practice toward BFHI

The results in Table 4 revealed that there is a statistically significant moderate positive relationship between knowledge and attitudes levels (*r* = 0.586, *p*-value <0.001). Meanwhile, there was a weak positive statistically significant relationship between Knowledge and practice levels (*r* = 0.279, *p*- value <0.001), and between attitudes and practice levels (*r* = 0.209, *p*-value <0.001).

**Table 4:** Correlation between knowledge, attitudes, and practice level.

Variable	Attitude (R)	Practice (R)
Knowledge	0.586**	0.279**
Attitude	–	0.209**

** Significant at *p*-value <0.001.

## 3 Discussion

This study assessed healthcare professionals' (HCPs) knowledge, attitudes, and practices (KAP) toward the Baby-Friendly Hospital Initiative (BFHI) in accredited and non-accredited hospitals in Jordan. The findings demonstrated that HCPs in accredited BFHI hospitals exhibited significantly higher knowledge scores (*p*-value <.05) compared with those in non-accredited facilities. This difference is likely attributable to the structured training, examinations, and systematic monitoring required by the Health Care Accreditation Council (HCAC) to ensure readiness for implementing the Ten Steps to Successful Breastfeeding.

These findings are consistent with Al-Qahtani et al., reported significantly higher agreement scores among nurses working in accredited hospitals compared with those in non-accredited institutions (17). Similarly, other studies have shown that accreditation enhances professional skills and fosters a culture of quality improvement through targeted training and accountability mechanisms (18). Collectively, this evidence supports the role of accreditation in strengthening healthcare providers' knowledge and preparedness to deliver quality maternal and neonatal care.

By contrast, no significant association was observed between hospital accreditation status and HCPs' attitudes or practices regarding BFHI. This suggests that while accreditation improves knowledge, it does not necessarily translate into changes in attitudes or everyday clinical practices (19). One possible explanation is that hospital policies and standardized methodologies are applied across facilities, thereby limiting observable differences. In addition, the specific roles and responsibilities of HCPs may not always involve direct implementation of BFHI practices, meaning that knowledge may not readily translate into behavior (19).

### 3.1 Knowledge, attitude, and practice regarding BFHI

In this study, the majority of healthcare professionals (HCPs) demonstrated a strong understanding of BFHI principles, with 92% correctly identifying the Ten Steps to Successful Breastfeeding. This finding is higher than reports from Nepal, where 58% of participants demonstrated adequate knowledge (20) and contrasts with results from Turkey, where only 28.5% of participants showed good knowledge (21). Such variation across countries may be explained by differences in hospital accreditation status, healthcare systems, and professional education levels.

With respect to attitudes, 63% of participants reported favorable views toward BFHI. This aligns with findings from Siyabulela and Leslie, (22) in South Africa, where nurses expressed positive attitudes toward BFHI implementation and its role in increasing breastfeeding rates (22).

Attitudes toward breastfeeding are often shaped by personal experiences, cultural influences, and professional confidence. Negative attitudes can hinder the application of new skills; if healthcare providers lack certainty in their guidance, they may struggle to support mothers effectively (23).

Conversely, mothers rely on professionals for encouragement, support, and practical advice (24).

Misconceptions, such as the belief that breastfeeding should be discontinued during mastitis, highlight the need for ongoing professional training. Evidence indicates that attitudes may not always align with actual practices, as staff can maintain less favorable personal views while still adhering to institutional policies (7).

Casal et al. reported that implementing baby-friendly practices on HCPs' breastfeeding attitudes had a positive effect, although it is not likely to determine which factors had the best association with modifying these attitudes (25). Further, Casal et al. concluded that attitudes are associated with people's emotions and feelings, and the success of these interventions relies on how successfully they affect emotions and whether these emotions changes impact people's behavior (25).

Moreover, interventions targeting emotions and beliefs appear most effective in shaping attitudes and subsequent behaviors (20). Similarly, a study found that 51% of respondents had poor practice (26)^.^ Such discrepancies likely reflect methodological differences across hospitals and varying institutional support for BFHI practices.

Importantly, strong positive correlations were observed between knowledge, attitudes, and practices (*p* < 0.001 for all associations). This indicates that higher knowledge of BFHI principles is linked to more favorable attitudes and better clinical practices (19). Conversely, inadequate knowledge may undermine both attitudes and behaviors. These findings are supported by previous studies demonstrating a similar relationship between knowledge, attitudes, and practice in breastfeeding promotion (8, 15).

### 3.2 Recommendations

To further enhance BFHI implementation, structured training programs should be provided regularly for all HCPs, with emphasis on correcting common misconceptions such as the management of breastfeeding during mastitis. Accreditation bodies and hospital administrations should ensure continuous professional development and supportive supervision to reinforce both knowledge and practice. Future research should explore barriers to translating knowledge into practice, particularly in non-accredited hospitals, and evaluate the long-term impact of accreditation on breastfeeding rates and infant health outcomes.

### 3.3 Strengths and limitations

This study is among the first in Jordan to directly compare the knowledge, attitudes, and practices of HCPs in accredited vs. non-accredited BFHI hospitals. The inclusion of multiple professional groups across several regions provides a broad representation of healthcare settings. The use of a validated questionnaire further strengthens the reliability of findings. On the other hand, several limitations should be considered. First, the study employed a cross-sectional design, which limits causal inference. The use of convenience sampling may have introduced selection bias, and self-reported questionnaires are subject to social desirability bias. Additionally, the study did not include university or private hospitals, which restricts the generalizability of the results to all healthcare facilities in Jordan.

## 4 Conclusion

Meeting the BFHI standards is a complex process that requires substantial changes in hospital policies, practices, and infrastructure. Despite these challenges, the findings of this study indicate that BFHI accreditation is associated with improved knowledge among HCPs, which in turn positively influences their attitudes and practices. Both accredited and non-accredited hospitals demonstrated generally good knowledge, but significant differences highlight the role of structured training and accreditation in strengthening BFHI implementation. These results underscore the importance of the hospital environment and healthcare providers' active role in promoting breastfeeding, which ultimately contributes to improved maternal and child health outcomes.

## Data Availability

The datasets presented in this study can be found in online repositories. The names of the repository/repositories and accession number(s) can be found in the article/Supplementary Material.
